# Evaluation of Perienhancing Area in Differentiation between Glioblastoma and Solitary Brain Metastasis

**DOI:** 10.31557/APJCP.2020.21.9.2525

**Published:** 2020-09

**Authors:** Jureerat Thammaroj, Nattha Wongwichit, Arunnit Boonrod

**Affiliations:** *Department of Radiology, Faculty of Medicine, Khon Kaen University, Khon Kaen, 40002, Thailand. *

**Keywords:** Perienhancing area, differentiation, glioblastoma, GBM, solitary brain metastasis, intratumoral necrosis

## Abstract

**Purpose:**

Accurate differential diagnosis between glioblastoma and brain metastasis is important. We aimed to differentiate these tumors by evaluation of the perienhancing area.

**Materials and Methods:**

Thirty patients with glioblastoma and solitary brain metastasis were included. The diameters of perienhancing and enhancing areas were measured, and the percentage of enhancing area was calculated. We measured Apparent diffusion coefficient (ADC) of perienhancing and enhancing areas. Intratumoral necrotic areas were measured.

**Results:**

The enhancing area of glioblastoma was 56.61% and metastasis was 42.55% (p = 0.08). The ADC values of the perienhancing part of GBM was 0.7 and metastasis was 0.79 (p = 0.052). The ADC value of the enhancing part of the GBM was 0.82 and metastasis was 0.8 (p-value = 0.72). The intratumoral necrotic area of glioblastoma (152.25 mm^3^) was higher than in metastasis (0 mm^3^) (p-value = 0.003) with a cutoff area of 11.8 mm^2^.

**Conclusion:**

The ADC values of the perienhancing area were lower in glioblastoma with a near-significant p-value. Other perienhancing parameters demonstrated no significant difference between both tumors. The intratumoral necrotic area of glioblastoma is larger than metastasis.

## Introduction

Glioblastoma and brain metastasis are two of the most common brain tumors in adults. Accurate imaging diagnosis is important because of the differing treatments for these conditions (Stark et al., 2012; Wu et al., 2015). Glioblastoma and solitary brain metastasis can sometimes appear similar on conventional MRI, which can make the definite diagnosis difficult. Both glioblastoma and brain metastasis demonstrate heterogeneous signal in conventional MRI with various types of enhancement surrounded by a perienhancing edematous area (Sentürk, Oğuz, and Cila, 2009; Hakyemez et al., 2010; Halshtok Neiman et al., 2013; Wu et al., 2015). 

Glioblastoma and metastasis differ in degrees of perilesional edema. Few previous studies have shown significant differences between the ratio of peritumoral edema and enhancing tumor (Maurer et al., 2013; Baris et al., 2016). Perilesional edema was greater in brain metastasis than in glioblastoma. 

Diffusion-weighted image (DWI) and apparent diffusion coefficient (ADC) values are known to help differentiate between tumor types because they represent the movement of water molecules in vivo (Hagmann et al., 2006; Phuttharak et al., 2018). Several studies have yielded inconclusive results when trying to make a differential diagnosis between glioblastoma and brain metastasis, especially when relying on values measured at the enhanced part of the tumor (Calli et al., 2006; Guzman et al., 2008; Lee et al., 2011; Tsougos et al., 2012; Maurer et al., 2013; Lemercier et al., 2014; Dawoud, Sherif, and Eltomey, 2014). Even though preliminary results are inconclusive, we believe DWI is a potential tool for differential diagnosis between these two tumors. In pathology, glioblastoma has combined peritumoral infiltration and edema but brain metastasis has pure perilesional vasogenic edema (Pekmezci and Perry, 2013; Louis et al., 2016). Accordingly, ADC values of the perilesional area should be different between these two groups: glioblastoma should have lower ADC values than metastasis. 

In our study, we evaluated two main aspects of the perihancing area. We measured the maximal diameter of the area and also the ADC values relative to the enhancing area, to differentiate between glioblastoma and solitary metastasis. 

## Materials and Methods

*Patients*

Thirty consecutive patients with histopathologically proven solitary brain metastasis (n=15) or glioblastoma (n=15) who underwent pre-treatment MRI of the brain using DWI, between 1 January 2011 and 1 December 2016, were retrospectively reviewed. One patient had two lesions pathologically proven to be glioblastoma: the total number of tumors included was therefore 31. The study was approved by the local Institutional Review Board of ethical issues with a waiver of informed consent.

*Imaging techniques*

Seven patients were imaged using a 1.5T MR scanner (MAGNETOM Aera; Siemens, Erlangen, Germany) and twenty-one patients were imaged with a 3T MR scanner (Phillips Achieva; Philips, Best, the Netherlands). Routine MR pulse sequences for the 1.5T scanner include: sagittal and axial T1-weighted image spin echo [T1WI SE] [TR 450-600, TE 8-10], axial T2-weighted image turbospin echo [T2WI turboSE] [TR 3500-5000, TE 80-100], axial fluid-attenuated inversion recovery [FLAIR] [TR 9000, TE 120, TI 2300], and coronal T2GRE [TR 700-800, TE 15-35, FA 20]. Routine MR pulse sequences for the 3T scanner include: sagittal T1WI 3D TFE [TR shortest, TE shortest, FA 8], axial T2WI TSE [TR 3000-5000, TE 80], axial FLAIR [TR 11000, TE 125, TI 2800], and coronal T2GRE [TR 700-800, TE 15-35, FA 18]. Imaging parameters of DWI were as follows: 1819-8000/85-93 [TR/TE] with diffusion sensitivities b=0 and b=1,000 s/mm^3^ for both scanners. The diffusion gradients were applied sequentially in three orthogonal directions to generate 2 sets of axial DW images. The ADC maps were automatically generated from the datasets of DW images using the operating console (Synapse 3D workstation, Fujifilm Medical Systems, USA) and ADC values were calculated. After administration of gadolinium-based contrast agent, multiplanar T1WI was performed. In all sequences, the field of view was 22-24 cm. and the section thickness was 5 mm. 

*Image analysis*

**Figure 1:**
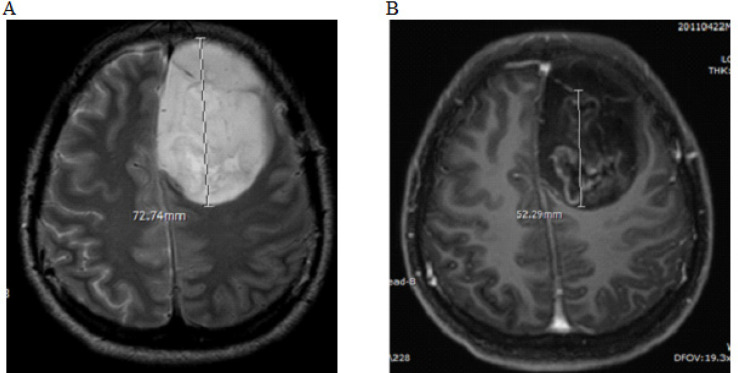
A 27-Year-Old Woman with a Glioblastoma at Left Frontal Lobe. T2WI (A) and post contrast T1WI (B) showed a heterogeneous hypersignal T2WI mass with irregular ring enhancement. The maximal diameters of perienhancing and enhancing areas were 72.7 and 52.3 mm, respectively. The percentage of enhancing area was 71.9%

**Figure 2:**
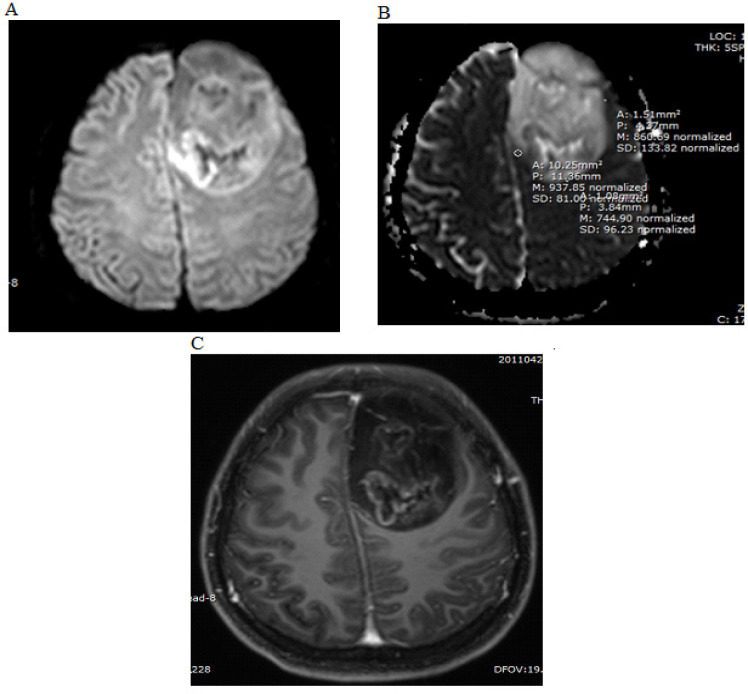
The Same Patient. DWI (A), ADC Map (B) and Axial Post Contrast T1WI (C) Demonstrated Restricted Diffusion Areas at Perienhancing Area. At this image, three ROIs were drawn on the ADC map (B) at the area with minimal ADC value. The measured ADC values in this image were 0.937, 0.744 and 0.86 x10^-3^mm^2^/s. The mean ADC value of the perienhancing area of this case was 0.86 x10^-3^mm^2^/s. Post-contrast image (C) showed that no enhancing part was included in the measurement of perienhancing area

All MRIs were analyzed by a senior neuroradiologist with 20 years’ experience in neuroimaging. We defined perienhancing areas as the non-enhancing hypersignal T2WI areas surrounding the enhancing tumor. At the perienhancing area, the maximal diameter in axial T2WI was measured, and the minimum ADC areas were visually inspected and five regions of interest (ROIs) were drawn in different places. At the enhancing area, the maximal diameter was measured in axial view on post-contrast T1WI and five ROIs for ADC values were measured (Figure 1 and 2). The percentage of the enhancing area to the perienhancing area was calculated. The ADC ratio was calculated by dividing the mean of five perienhancing ADC values by the mean of five enhancing ADC values.

**Figure 3:**
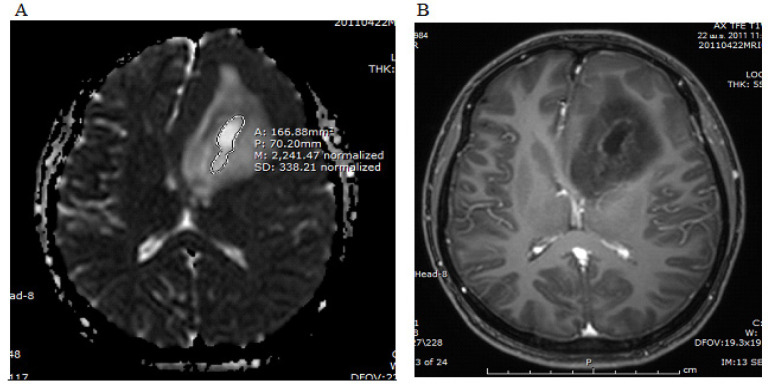
The Same Patient, ADC Map (A) and Post Contrast T1WI (B) Demonstrated an Intratumoral Non-Enhancing Area with Increased Diffusion, Representing the Intratumoral Necrotic Area. ROIs were drawn free-hand on the ADC map. The intratumoral necrotic area in this image was 166.9 mm^2^ and the sum of intratumoral necrotic area measurements in this patient was 561.4 mm^2^

We also recorded the extent of intratumoral necrotic areas (mm^2^). We defined these areas as the non-enhancing hypersignal T2WI area within the enhancing tumor if they showed increased diffusion on DWI and ADC maps (hypersignal on both DWI and ADC maps). The area was calculated from free-hand ROI drawings on the ADC map (Figure 3) If more than one necrotic area was identified, each area was measured at its largest extent and values were summed. 

*Statistical analysis*

Statistical analysis was performed using Stata statistical software package, version 10. Demographic data of all patients and the site of the primary tumor of the metastasis patients were interpreted by descriptive analysis. To determine the difference between metastasis and glioblastoma, we analyzed the ratios of diameters, the ADC values, and ADC ratios using two-sample Wilcoxon rank-sum tests (Mann-Whitney). A receiver operating characteristic (ROC) analysis was used to determine the cutoff value, sensitivity and specificity of the intratumoral necrotic area as a tool for differentiation between glioblastoma and brain metastasis. A p-value of < 0.05 indicated a statistically significant difference.

## Results

Of the thirty patients included, sixteen were males (53.3%) and fourteen were females (46.7%). Their ages ranged from 7 to 71 years (48±18.5 years, mean±SD). Both glioblastoma and metastasis groups had fifteen patients, eight males and seven females. The mean age of metastasis patients was 58 (±10) years and the mean age of glioblastoma patients was 38 (±20) years. The primary tumors producing solitary metastasis were lung cancer (7 patients, 46.7%), head and neck cancer (3 patients, 20%), breast cancer (2 patients, 13.3%), neuroendocrine tumor (1 patient, 6.7%), renal angiomyelolipoma (1 patient, 6.7%), and cholangiocarcinoma (1 patient, 6.7%).

Table 1 provides summary statistics (medians and percentiles) on the extent of enhancing areas, ADC values of the perienhancing and enhancing areas (Figure 1 and 2), ADC ratios (Figure 2) and intratumoral necrotic areas (Figure 3) of the glioblastoma and metastatic groups. The median of ADC values (x10^-3^mm^2^/s,) of perienhancing areas of glioblastomas and metastasis were 0.7 and 0.79 (p-value = 0.052). The median of ADC values (x10^-3^mm^2^/s,) of enhancing areas of glioblastomas and metastasis were 0.82 and 0.8 (p-value = 0.72). The median of ADC ratios, calculated by dividing the perienhancing ADC value by the enhancing area ADC value, of glioblastomas and metastasis were 0.87 and 0.95 (p-value = 0.52). The median of intratumoral necrotic areas (mm^2^) of glioblastomas and metastasis were 152.25 and 0 (p-value = 0.003).

**Figure 4:**
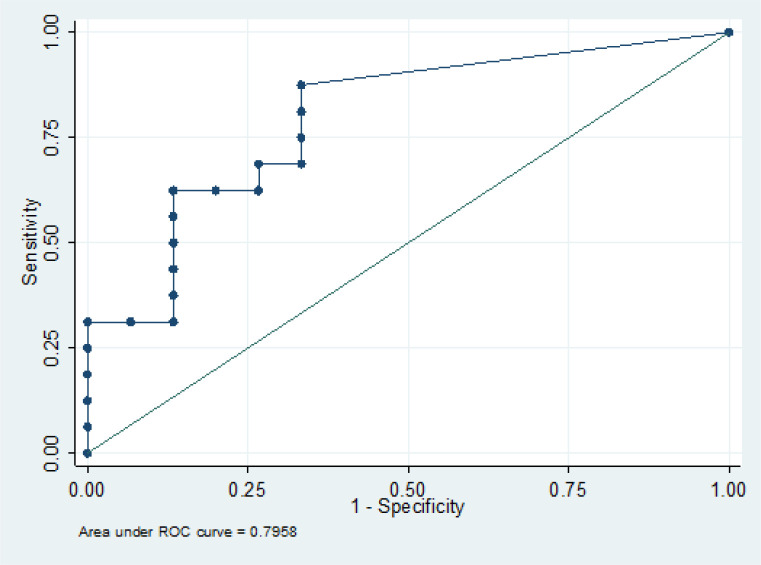
Receiver Operating Characteristic (ROC) Curve of Intratumoral Necrotic Area for Differentiation of Glioblastoma from Metastasis. The area under the ROC curve was 0.8 (95% CI 0.64, 0.95). When the cutoff necrotic area was set as 11.8 mm^2^, sensitivity (87.5%) and specificity (66.67%) were obtained

A receiver operating characteristic (ROC) curve was used to analysis the intratumoral necrotic area for differentiation of glioblastoma and metastasis. The area under the ROC curve was 0.80 (95% CI 0.64, 0.95). When the cutoff value for the necrotic area was set as 11.8 mm^2^, sensitivity (87.5%) and specificity (66.67%) were obtained (Figure 4).

**Table 1:** Comparisons between Glioblastoma and Metastasis in Terms of ADC Values, ADC Ratios, Intratumoral Necrotic Areas and Percentage of Enhancing Areas

	Median	P25,P75	p-value
Perienhancing ADC values ( x10^-3^mm^2^/s)			
GBM	0.7	0.59, 0.76	0.052*
Metastasis	0.79	0.74, 0.92	
Enhancing ADC values ( x10^-3^mm^2^/s)			
GBM	0.82	0.73, 0.94	0.72
Metastasis	0.8	0.72, 1.11	
ADC ratio^ a^			
GBM	0.87	0.67, 1.05	0.52
Metastasis	0.95	0.73, 1.03	
Area of tumoral necrosis (mm^2^)			
GBM	152.25	28.2, 362.9	0.003**
Metastasis	0	0, 103.6	
Percentage of enhancing area			
GBM	56.6	48.5, 74.3	0.08
Metastasis	42.6	29.6, 66.7	

^a ^ADC ratio was calculated by dividing the perienhancing ADC value by the enhancing area ADC value; *, Near significant p-value; **, Significant p-value

## Discussion

Differentiation between glioblastoma and brain metastasis is significant for the planning of both diagnostic workup and treatment. Accurate pre-treatment diagnosis will be a benefit to both the patients and the clinicians. 

Heterogeneous cell structures were found in the solid tumoral parts of both glioblastoma and metastasis tissues in histology. However, at the peritumoral area, a glioblastoma typically has peritumoral infiltration, but a brain metastasis does not (Pekmezci and Perry, 2013). Several studies have shown no significant difference in peritumoral ADC values between glioblastoma and metastases (Calli et al., 2006; Lee et al., 2011; Maurer et al., 2013; Dawoud et al., 2014). Few studies have shown lower peritumoral ADC values in glioblastoma as compared to metastasis (Guzman et al., 2008; Lemercier et al., 2014; Blystad et al., 2017). We found median ADC values in the non-enhancing area at the edge of the tumor to be lower in metastases than in glioblastoma, with a near-significant p-value (p-value = 0.052). This finding may help to distinguish between GBM and metastases and supports the hypothesis that a glioblastoma involves peritumoral infiltration but a brain metastasis does not (Chilla et al., 2015; Celik, 2016).

An enhancing mass with extensive perilesional brain edema is a classic characteristic of brain metastasis. However, the perilesional edema in metastasis can vary from minimal to extensive (Sharma et al., 2013). A previous study found a statistically significant difference in the ratio of enhancing tumor to perilesional edema (Maurer et al., 2013; Baris et al., 2016), whereas we found no such difference.

Intratumoral necrosis is believed to occur because of insufficient blood supply within a rapidly growing malignant tumor. However, for glioblastoma, microvascular hyperplasia is found around the necrotic area, which may represent an prothrombotic or vaso-occlusive state within the tumor (Altmanet al., 2007) This may cause more intratumoral necrosis in glioblastoma as compared to brain metastasis, as reflected in the significant difference in our study. The intratumoral necrotic area in glioblastoma was higher than in metastases (p-value = 0.003) with the cutoff point of 11.8 mm^3^ (sensitivity of 87.5% and specificity of 66.67%). 

This study had a few limitations. First, the study was retrospective in nature. Second, two different MR scanners (1.5T and 3T) were used, with slightly different technique, which may reduce comparability of the ADC values. Third, the sample size was small with a considerable age difference between the glioblastoma and metastasis group. Further prospective study with larger populations and uniform MR technique are required to extend the results.

In conclusion, the ADC values of the perienhancing area were lower in glioblastoma than in solitary brain metastases with a near-significant p-value. Other perienhancing parameters, including ADC ratio and percentage of enhancement, demonstrated no significant differences between both tumors. The intratumoral necrotic area of glioblastoma is larger than metastasis with the cutoff value of 11.8 mm^2^.
